# Protectin DX Attenuates Lumbar Radicular Pain of Non-compressive Disc Herniation by Autophagy Flux Stimulation *via* Adenosine Monophosphate-Activated Protein Kinase Signaling

**DOI:** 10.3389/fphys.2021.784653

**Published:** 2022-01-06

**Authors:** Qing-xiang Zhao, Yi-hao Wang, Si-cong Wang, Song Xue, Zhen-xin Cao, Tao Sun

**Affiliations:** ^1^Department of Pain Management, Shandong Provincial Hospital, Cheeloo College of Medicine, Shandong University, Jinan, China; ^2^Department of Pain Management, Binzhou Medical University Hospital, Binzhou, China; ^3^Departments of Pain Management, Shandong Provincial Hospital Affiliated to Shandong First Medical University, Jinan, China

**Keywords:** lumber radicular pain, adenosine monophosphate-activated protein kinase, inflammatory mediator, protectin DX, autophagy flux

## Abstract

**Background:** Neuroinflammation plays a crucial role in initiating and sustaining lumbar radicular pain (LRP). Protectin DX (PDX) has been experimentally verified to possess pro-resolving properties and anti-inflammatory effects. This study aimed to observe the analgesic effects of PDX and its potential mechanisms in LRP rats with non-compressive lumbar disc herniation (NCLDH).

**Method:** Only male rats were selected to avoid gender-related interferences. Rat models of NCLDH were established, and rats were randomly divided into four groups: the sham group, the vehicle group, the PDX (10 ng PDX) group, and the PDX (100 ng PDX) group. Changes in the mechanical withdrawal threshold and thermal withdrawal latency were observed for 7 days. The mRNAs of pro-inflammatory and anti-inflammatory mediators were evaluated *via* real-time polymerase chain reaction, whereas western blot and immunohistochemistry were separately conducted to assess the expression levels of autophagy-related proteins and adenosine monophosphate-activated protein kinase (AMPK) signaling.

**Results:** Intrathecal delivery of PDX reduced interleukin (IL)-6 and IL-1β mRNA levels and facilitated mRNA transcription of transforming growth factor-β_1_, with attenuation of mechanical and thermal hyperalgesia in LRP rat models. With the application of nucleus pulposus to the dorsal root ganglion, autophagy flux and AMPK signaling were severely disrupted in the spinal dorsal horns, and intrathecal treatment with PDX could dose-dependently restore the dysfunction of autophagy flux and AMPK signaling.

**Conclusion:** These data suggest that PDX possesses pro-resolving properties and exerts potent analgesic effects in LRP by affecting autophagy flux *via* AMPK signaling.

## Introduction

Lumbar radicular pain (LRP), commonly secondary to protruded nucleus pulposus (NP), has been cited as the most frequent form of chronic pain disorder (Jensen et al., 2019). Based on epidemiological data, with the point prevalence exceeding 13.4% (Konstantinou and Dunn, 2008), LRP was estimated to have a lifetime prevalence of up to 43% in the general adult population (Hofmann et al., 2002). Formerly, a wealth of clinical evidence supported the theory that mechanical compression was frequently associated with the pathophysiology of LRP. At present, however, increasing preclinical studies (Huang et al., 2018a; Wang et al., 2020) have revealed that neuroinflammation caused by NP could directly provoke LRP, even with a lack of nerve root compression. Clinically, the presumption of inflammation on the pathophysiology of LRP has also presented an effective treatment strategy for this disorder (Kennedy et al., 2018). However, drugs targeting inflammation, specifically nonsteroidal anti-inflammatory drugs or corticosteroids, have displayed restricted efficacy and undesired side effects on patients suffering from LRP (Goldberg et al., 2015; Markman et al., 2018).

The most recent studies indicate that subsidence of inflammation was dominated by endogenous substances collectively named special pro-resolving lipid mediators (SPMs), and failed resolution could markedly aggravate autoinflammatory or autoimmune diseases (Dalli and Serhan, 2019; Sugimoto et al., 2019). Protectin DX (PDX), synthesized from docosahexaenoic acid, is a novel member of SPMs and has been observed to possess potent pro-resolving properties in acute and chronic inflammation (White et al., 2014; Sanceau et al., 2019). Furthermore, it has been demonstrated that PDX could hinder the development of inflammation in several inflammation-related disorders, such as osteoarthritis (Piao et al., 2020) and sepsis (Xia et al., 2020). However, little research has been conducted on whether PDX could exert anti-inflammatory actions and analgesic properties in LRP. In addition, PDX has recently been identified as a novel adenosine monophosphate-activated protein kinase (AMPK) activator, and emerging studies indicate (Herzig and Shaw, 2018; Clarke and Simon, 2019; Levine and Kroemer, 2019) that AMPK activation is interrelated with autophagy regulation, which is critically involved in the pathophysiology of chronic pain disorders. Therefore, we hypothesized that the pro-resolving and anti-inflammatory property of PDX might be linked to AMPK-mediated autophagy.

Hence, the objective of this research was to observe the analgesic effects of exogenous PDX in LRP rats with non-compressive lumbar disc herniation (NCLDH). Furthermore, to assess the underlying mechanisms, we also evaluated autophagy flux and AMPK signaling in rats with LRP by utilizing histological and protein analysis.

## Materials and Methods

### Animals

Adult male Sprague–Dawley rats (260–300 g) were obtained from the Experimental Animal Center of Beijing Vital River (Beijing, China). All rats were housed separately at a constant temperature of 23°C ± 1°C, and 12:12 h light and dark cycle. During the experiment, the rats had free access to water and standard rodent chows. All animal experiments were sanctioned by the Animal Care and Use Committee at Shandong University.

### Surgical Procedures

Surgical procedures were performed under sodium pentobarbital anesthesia (40 mg/kg, i.p.). Polyethylene catheters (PE-10; Smiths Medical, United Kingdom) for intrathecal delivery were implanted as described by Shih et al. (2012). After L4–L5 vertebrae were fully exposed by detaching back muscles, PE-10 was cautiously inserted into subarachnoid space until cerebrospinal fluid outflow. Passed through the subcutaneous tissue, the external PE-10 was firmly immobilized at head. Three days after catheter implantation, animals were observed: only rats without neurological deficit were used to establish the rat models.

Lumbar radicular pain rat models of NCLDH were carefully established, according to (Zhang et al., 2011; Liu et al., 2019). After exposure of the right L5 dorsal root ganglion (DRG), the equivalent NP from two tail intervertebral discs of the same animal was implanted on right L5 DRG with great care, to avoid compression. Except for the placement of the NP, the same procedure was conducted for the sham group.

### Intrathecal Drug Delivery

Once the rat models were successfully established, intrathecal delivery was conducted through the implanted PE-10 catheters. The hind limbs of the rats were paralyzed by intrathecal delivery of 2% lidocaine (10 μl) and the location of catheterization was ascertained to be successful. After removal of the ethanol following the manufacturer’s instructions, PDX (Cayman Chemical Company, United States) was fully dissolved in sterile phosphate buffer saline (PBS). Subsequently, PDX (10 ng or 100 ng; 10 μl) or vehicle (equivalent PBS) were intrathecally injected into the rats. All rats received either the drug or the vehicle on postoperative days 1, 2, and 3.

### Measurement of Pain-Related Behavior

Before the behavioral measurement, rats were individually acclimated to the testing conditions for 0.5 h. As described previously (Hargreaves et al., 1988; Chaplan et al., 1994), thermal withdrawal latency (TWL) was evaluated with a BME-410A thermal dolorimeter, and mechanical withdrawal threshold (MWT) was detected *via* von Frey up-down testing. These measurements were obtained on preoperative day 1 and postoperative days 1–7, respectively. Handlers were blinded to the drug delivery of rats.

### Quantitative Real-Time Polymerase Chain Reaction

On postoperative day 7, deeply anesthetized rats were euthanized *via* exsanguination of the aorta. Tissues of ipsilateral spinal dorsal horn (L4–L6) were cautiously detached for real-time polymerase chain reaction (RT-PCR) and immunoblot analysis. Following the manufacturer’s recommendation, t-RNA was harvested by utilizing the TRIzol method (Takara, Dalian, China), and cDNA was synthesized by the PrimeScript RT reagent kit (Takara, Dalian, China). The amplifications of PCR were then utilized in Light Cycler® 480 II (Roche, Switzerland) by SYBR Green Premix Ex Taq (Takara, Dalian, China). The primers were sourced from BioSune (China) and applied as follows (Table 1). Ultimately, normalized with β-actin, mRNA levels were evaluated utilizing the 2^−ΔΔ*C*t^ method.

**Table 1:** Primer sequence for RT-PCR.

Gene	Primer	Sequences
β-actin	Forward	5ʹ-CCCATCTATGAGGGTTACGC-3ʹ
	Reverse	5ʹ-TTTAATGTCACGCACGATTTC-3ʹ
IL-Iβ	Forward	5ʹ-GAGGACCCAAGCACCTTCT-3ʹ
	Reverse	5ʹ-CCGTCTTTCATCACACAGGA-3ʹ
IL-6	Forward	5ʹ-AAGTCGGAGGCTTAATTACATATGTTC-3ʹ
	Reverse	5ʹ-TGCCATTGCACAACTCTTTTCT-3ʹ
TGF-β1	Forward	5ʹ-ATTCCTGGCGTTACCTTGG-3ʹ
	Reverse	5ʹ-AGCCCTGTATTCCGTCTCCT-3ʹ

*RT-PCR, real-time polymerase chain reaction; IL, interleukin; TGF-β1, transforming growth factor-β1*.

### Western Blot Analysis

Following the manufacturer’s protocol, tissue proteins of ipsilateral spinal dorsal horn were isolated using RIPA lysis buffer (Beyotime Biotechnology, CHN). To separate proteins of 30 μg sample, electrophoresis was utilized through 8–15% polyacrylamide gels. Proteins were electrotransferred to PVDF membranes (Millipore, United States), which were then blocked with 5% nonfat milk. The following primary antibodies were applied overnight at 4°C: mouse anti-GAPDH antibody (sc-32233; 1:500; Santa, United States), rabbit anti-LC3B antibody (18725-1-AP; 1:1,000; Proteintech, CHN), rabbit anti-P62 antibody (66184-1-Ig; 1:5,000; Proteintech, CHN), rabbit anti-p-AMPK antibody (2535; 1:1,000; CST, United States), and rabbit anti-AMPK antibody (5832, 1:1,000; CST, United States). The following secondary antibodies were applied for 60 min at room temperature: HRP-conjugated goat anti-rabbit (BA1054; 1:5,000; Boster, CHN) and HRP-conjugated goat anti-mouse (BA1050; 1:5,000; Boster, CHN). An enhanced chemiluminescence assay (Millipore, United States) was conducted to develop the protein bands, and densities of target proteins were quantified relative to GAPDH using Image-Pro Plus software.

### Immunohistochemistry

On postoperative day 7, tissues of ipsilateral spinal dorsal horn (L4–L6) were cautiously detached and immediately immersion fixed overnight in 4% paraformaldehyde. Thereafter, a series of steps were performed as manufacturer’s instructions (ZSGB, CHN), including dehydrating, embedding, dissecting (sectioned at 4 μm), dewaxing, and rehydration. Following antigen retrieval in the citrate buffer (ZLI-9064, ZSJQ) under high-temperature and high-pressure for 2 min, the sections were then inactivated for endogenous peroxidase for 30 min at 37°C. The sections were processed overnight with rabbit anti-LC3B (18725-1-AP; 1:500; Proteintech, CHN), rabbit anti-P62 (66184-1-Ig; 1:2,000; Proteintech, CHN), and rabbit anti-phospho-AMPK (2535; 1:200; CST, United States) at 4°C, and incubated with goat anti-rabbit secondary antibody (PV-9000; 1:100; ZSGB, CHN) for 30 min at 37°C. Immunopositive reactions were observed using a diaminobenzidine kit (ZSGB, CHN), followed by hematoxylin staining and sealing. Pictures were captured with a Leica DM4000B microscope and a digital camera (Germany). The integrated optical density (IOD) of positive reactions was quantified relative to the area (entire dorsal horn) using Image-Pro Plus software.

### Statistical Analysis

Data are presented as the mean ± SEM. Statistical computations were conducted using SPSS 21.0 software (IBM, United States) and results were graphed using GraphPad Prism®8.02 (GraphPad Software, United States). All data reached the normal distribution except for MWT, which was log10 transformed. Behavioral data of rats were processed using a two-way analysis of variance (ANOVA), whereas differences among biochemical data obtained *via* RT-PCR, western blot, and immunohistochemistry were determined with one-way ANOVA, followed by Tukey’s multiple comparisons test. *p* < 0.05 was rated an acceptable level of significance.

## Results

### Protective Effects of PDX in LRP Rat Models of NCLDH

**Figure 1:**
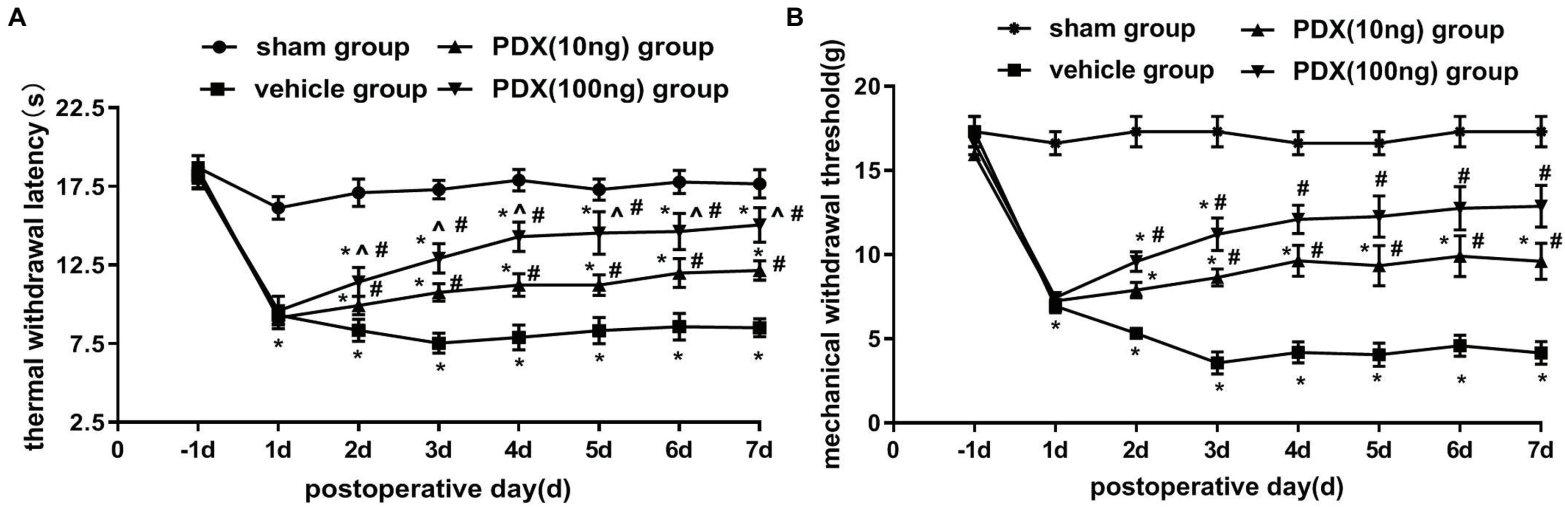
Protective effects of protectin DX (PDX) in lumbar radicular pain (LRP) rat models of non-compressive lumbar disc herniation (NCLDH; *n* = 9/group). **(A)** Thermal withdrawal latency (TWL) was evaluated with BME-410A thermal dolorimeter, **(B)** mechanical withdrawal threshold (MWT) was detected by applying von Frey up-down testing. PDX (10 ng or 100 ng) prominently meliorated the TWL and MWT of LRP rat models. Two-way ANOVA was used for comparisons among groups followed by Turkey’s multiple comparison test (log10 transformation for the statistical analysis of the MWT); Data are presented as the mean ± SEM (^*^p < 0.05 vs. sham group; ^#^*p* < 0.05 vs. vehicle group; ^*p* < 0.05 vs. 10 ng group).

Changes in pain-related behavior with intrathecal PDX treatment are shown in Figures 1A,B. After the transplant of NP to DRG, MWT and TWL were markedly decreased in the vehicle group; this lasted for at least 7 days (*p* < 0.05). Relative to the vehicle group, PDX (10 ng group or 100 ng group) significantly increased MWT and prolonged TWL (*p* < 0.05). Furthermore, the data showed that there was a dose-dependent relationship between TWL and PDX in LRP rat models (*p* < 0.05).

### PDX Prominently Regulates IL-6 and IL-1β mRNA Levels and Transforming Growth Factor-β_1_ in Spinal Dorsal Horns

**Figure 2:**
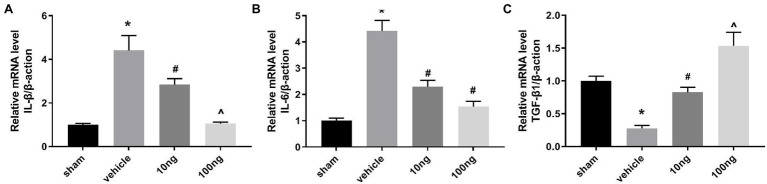
Protectin DX prominently regulated the mRNA productions of cytokines (*n* = 5/group). **(A–C)** Real-time polymerase chain reaction (RT-PCR) were executed to measure the inflammatory cytokines’ mRNA levels. Intrathecal delivery of PDX (10 ng or 100 ng) suppressed the mRNA levels of IL-1β and IL-6, and facilitated the mRNA expressions of TGF-β_1_. One-way ANOVA was used for comparisons among groups followed by Turkey’s multiple comparison test; Data are presented as mean ± SEM (^*^p < 0.05 vs. sham group; ^#^*p* < 0.05 vs. vehicle group; ^^^*p* < 0.05 vs. 10 ng group).

For detecting mRNA productions of IL-6, IL-1β, and transforming growth factor (TGF)-β_1_, RT-PCR was performed on ipsilateral spinal dorsal horns (Figures 2A–C). With exposition of NP to DRG, IL-6 and IL-1β mRNA productions were markedly induced; in contrast, mRNA productions of TGF-β_1_ were restricted in the vehicle group (*p* < 0.05). Intrathecal delivery of PDX (10 ng or 100 ng) diminished IL-6 and IL-1β mRNA levels and facilitated mRNA expressions of TGF-β_1_ (*p* < 0.05). Furthermore, the data showed that there was a dose-dependent relationship for IL-1β mRNA and TGF-β_1_ levels. These data indicated that intrathecal delivery of PDX could exert analgesic actions by governing the imbalance of cytokines in LRP rat models.

### PDX Facilitated Autophagy Flux in a Dose-Dependent Manner

**Figure 3:**
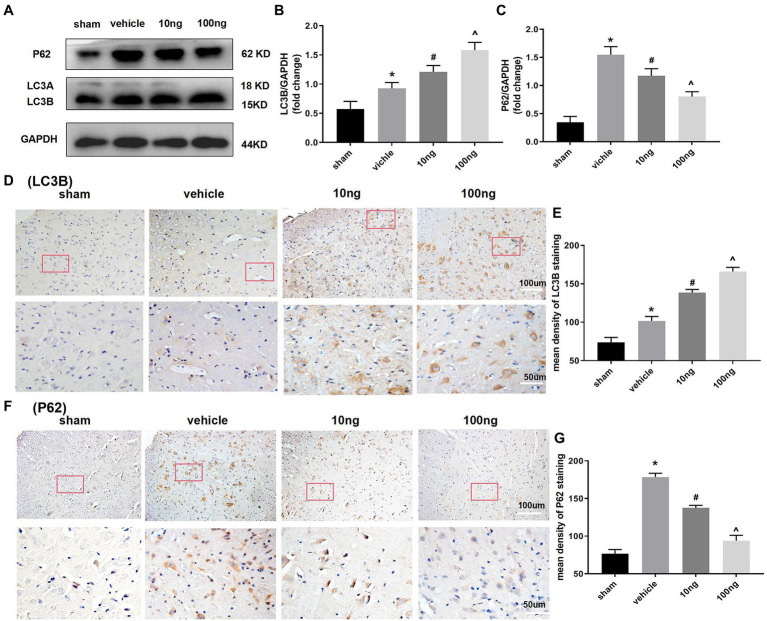
Protectin DX dose-dependently facilitated autophagy flux in spinal cord horns (*n* = 3/group). Western blot **(A)** and immunohistochemistry **(D,F)** were respectively conducted to determine the protein levels of expression of LC3B and p62. The intrathecal treatment of PDX could dose-dependently boost protein levels of LC3B and inhibit protein expression of P62. Statistical analysis of LC3B **(B,E)** and p62 **(C,G)**. One-way ANOVA was used for comparisons among groups followed by Turkey’s multiple comparisons test; Values are displayed as mean ± SEM (^*^p < 0.05 vs. sham group; ^#^*p* < 0.05 vs. vehicle group; ^^^*p* < 0.05 vs. 10 ng group).

Western blots were performed to monitor autophagy-associated protein in the spinal dorsal horns (Figures 3A–C). Relative to the sham group, significantly higher LC3B and P62 protein levels were displayed in the vehicle group (*p* < 0.05), implying the autophagy flux was hindered in the spinal dorsal horns. Treatment with PDX dose-dependently boosted protein levels of LC3B and inhibited protein expression of P62 (*p* < 0.05), which indicated that PDX might effectively facilitate autophagy flux in LRP rat models. Immunohistochemistry analysis further confirmed these results (Figures 3D–G).

### Effect of PDX on AMPK Signal Pathway

**Figure 4:**
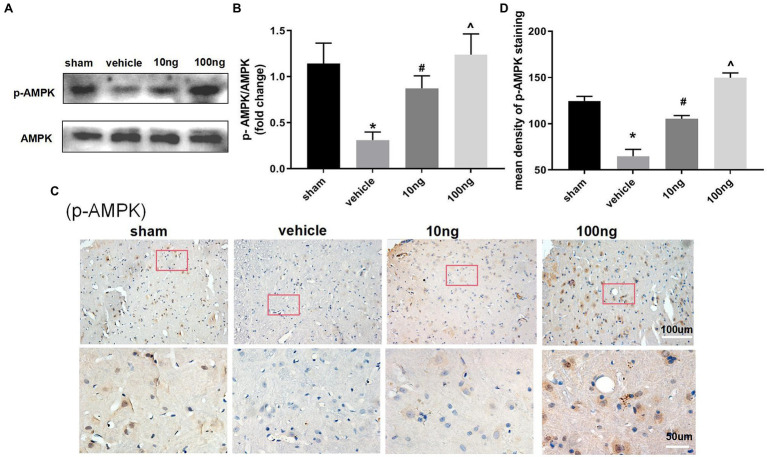
Effect of PDX on adenosine monophosphate-activated protein kinase (AMPK) signal pathway in spinal cord horns (*n* = 3/group). Western blot **(A)** and immunohistochemistry **(C)** were respectively conducted to evaluate the alterations in the AMPK signaling pathway. Relative to sham group, the protein levels of expression of p-AMPK markedly were suppressed in model rats. Meanwhile, the treatment of PDX could dose-dependently reverse the upper alterations of p-AMPK. Statistical analysis of p-AMPK **(B,D)**. One-way ANOVA was used for comparisons among groups followed by Turkey’s multiple comparisons test; Values are displayed as mean ± SEM (^*^p < 0.05 vs. sham group; ^#^*p* < 0.05 vs. vehicle group; ^^^*p* < 0.05 vs. 10 ng group).

To further determine the underlying mechanisms of PDX actions on autophagy flux and pro-resolution, western blotting (Figures 4A,B) and immunohistochemistry (Figures 4C,D) were separately conducted to detect changes in AMPK signaling. Relative to the sham group, p-AMPK/AMPK were distinctly suppressed in the vehicle group (*p* < 0.05). Treatment with PDX dose-dependently reversed the upper alterations (*p* < 0.05), indicating that PDX might partly facilitate autophagy flux *via* AMPK signaling in LRP rat models. Furthermore, immunohistochemistry analysis was consistent with the western blot results (Figure 4D).

## Discussion

This study found that intrathecal delivery of PDX notably suppressed IL-6 and IL-1β mRNA levels, and significantly facilitated mRNA transcription of TGF-β_1_, with the attenuation of hyperalgesia in LRP rats. With the transplant of NP to DRG, autophagy flux and AMPK signaling were reduced in the spinal dorsal horns, and PDX treatment dose-dependently restored dysfunction of autophagy flux and AMPK signaling in LRP rat models.

Neuroinflammation has been ascribed to the pathophysiology of LRP following disc herniation, in conjunction with mechanical components (Dower et al., 2019). Indeed, decades of clinical observations (Pedersen et al., 2015; Monchaux et al., 2017) have clarified that the severity of hyperalgesia is not closely connected with the size of disk herniation and that cytokines play a critical role in initiating and sustaining LRP. The latest study (Huang et al., 2018b) demonstrated that NP contact, as an irritant to the immune system, could stimulate spontaneous inflammatory firing in the neural system, leading to sensitization of nociceptive pathways. Thus, the LRP models of NCLDH have received growing attention in recent times. Our study also showed that after surgery, MWT and TWL distinctly decreased in the ipsilateral paw of the NCLDH models from the first day to the third day, and maintained until the final day of the experiment. Only male rats were selected to avoid gender-related interferences.

It has been demonstrated that an imbalance between anti-inflammatory and pro-inflammatory cytokines in the neural system is closely associated with the provocation and fueling of diverse neuropathic pain. In fact, among the cytokines, Interleukin (IL)-β and IL-6 have been considered vital molecules contributing to pain-related behaviors (Pedersen et al., 2015). Anti-inflammatory factors, such as TGF-β1, possibly produce a significant analgesic effect in pain conditions (Echeverry et al., 2009; Chen et al., 2013). We were curious as to whether this imbalance existed in LRP. Our current results show that in LRP rat models, high levels of IL-6 and IL-1β mRNA were induced, and expression of TGF-β1 mRNA was decreased, which was accompanied by a remarkable decrease in pain threshold.

Protectin DX is described as a newly identified SPM (Sanceau et al., 2019). Emerging studies revealed that PDX could facilitate the resolution of inflammation by driving macrophage emigration, phagocytosis, and M2 polarization (Xia et al., 2017; Ye et al., 2020). Moreover, PDX has been found to control progressive inflammation by suppressing platelet aggregation and preventing leukocyte infiltration and oxidative stress (Stein et al., 2016; Hwang et al., 2019). Only one research (Fonseca et al., 2017), found that it did not produce anti-nociceptive effect, which indicates that the roles of PDX might vary in different model selection or differed administration. In our study, intrathecal administration of PDX (10 ng or 100 ng) suppressed mRNA expression of IL-6 and IL-1β and facilitated mRNA translation of TGF-β_1_, with attenuation of hyperalgesia in LRP rat models. This difference may also be due to the method for assessing mechanical sensitivity. Differing fundamentally from Randall-Selitto test, von Frey filaments performed in our study may also activate low-threshold mechanoreceptors (Deuis et al., 2017). Thus, intrathecal delivery of PDX could induce analgesic actions by governing the imbalance of cytokines in LRP rat models.

The underlying mechanism of PDX involved in pro-resolving properties and analgesic actions has not been comprehensively explored. The activation of autophagy might be involved. In fact, because of the unique functions in eliminating damaged mitochondria and mitigating harmful substances through the lysosomal degradation pathway, autophagy was deemed (Clarke and Simon, 2019) to be a fundamental cellular mechanism in counteracting various stressors. Recent investigations (Areti et al., 2017; Weng et al., 2019) have shown that this pivotal feature made autophagy dysfunction result in diverse pain-associated diseases, for instance, oxaliplatin-evoked pain, osteoarthritis-related pain, and painful diabetic neuropathy. However, researchers seldom investigated the mechanisms behind PDX and LRP.

Additionally, autophagy has been identified as a dynamic process from the formation of autophagosomes to cargo degradation, which is termed autophagy flux. LC3B, a pivotal protein in autophagic substrate selection, is widely considered the most experimentally straightforward marker for detecting the autophagic process (Moulis and Vindis, 2017). However, accumulation of LC3B could indicate either the initiation of autophagosome biogenesis or a disruption of the degradation process. Given these potential limitations, to accurately track autophagic flux, emerging evidence (Kim et al., 2020) suggests that the biochemical analysis of LC3B should be conducted in combination with p62, which is efficiently degraded in the lysosome through the autophagy pathway. In our study, compared with sham group, significantly higher levels of LC3B and P62 proteins were observed in the vehicle group, indicating that autophagy flux was hindered in the spinal dorsal horns. Furthermore, PDX administration dose-dependently reversed the disrupted autophagy flux in LRP rat models.

A growing body of evidence (Piao et al., 2020; Yang et al., 2020) indicates that the pro-resolving effects of PDX are well correlated with AMPK signaling. The latest studies (Liu et al., 2019; Xiang et al., 2019) have also confirmed that activated AMPK signaling exerts protective properties in chronic pain including LRP. Furthermore, the majority of studies have shown that AMPK phosphorylation markedly induced autophagy flux. Our research suggested that relative to the sham group, p-AMPK/AMPK were markedly reduced in the vehicle group. The intrathecal delivery of PDX could dose-dependently reverse the impaired AMPK signaling in LRP rat models.

Finally, it remains to be investigated whether regulation of autophagy flux could directly affect inflammatory profile in LRP rat models. Recent studies have found (Cadwell, 2016; Deretic and Levine, 2018) that dysregulation of autophagy was closely related to neuroinflammation, and that autophagy activation could promote the pro-resolution of the inflammatory response. Also, it is yet to be determined whether PDX would have analgesic effect if later time points for administration.

In conclusion, our findings indicated that the imbalance between anti-inflammatory and pro-inflammatory cytokines in the central nervous system was closely correlated with the initiation and development of LRP. Mitigation of the above imbalance could be governed by intrathecal delivery of PDX, leading to the attenuation of hyperalgesia in LRP rat models. Moreover, the analgesic effects and pro-resolving properties of PDX in LRP rat models might be mediated, at least partially, by the AMPK-mediated autophagy flux. These findings may provide an interesting new insight into treating LRP.

## Data Availability Statement

The original contributions presented in the study are included in the article/supplementary material, further inquiries can be directed to the corresponding author.

## Ethics Statement

The animal study was reviewed and approved by the Animal Care and Use Committee at Shandong University.

## Author Contributions

QZ took the primary role in carrying out this work. YW and SW helped establish the rat models. SX and ZC helped implement the behavior testing. TS contributed to the conception and design of this study and modified the manuscript. All authors contributed to the article and approved the submitted version.

## Funding

This study received financial support from National Natural Science Foundation of China (Grant Nos. 81772443 and 81972145).

## Conflict of Interest

The authors declare that the research was conducted in the absence of any commercial or financial relationships that could be construed as a potential conflict of interest.

## Publisher’s Note

All claims expressed in this article are solely those of the authors and do not necessarily represent those of their affiliated organizations, or those of the publisher, the editors and the reviewers. Any product that may be evaluated in this article, or claim that may be made by its manufacturer, is not guaranteed or endorsed by the publisher.
